# Effect of home-based specialised palliative care and dyadic psychological intervention on caregiver anxiety and depression: a randomised controlled trial

**DOI:** 10.1038/s41416-018-0193-8

**Published:** 2018-11-14

**Authors:** Annika von Heymann-Horan, Pernille Bidstrup, Mai-Britt Guldin, Per Sjøgren, Elisabeth Anne Wreford Andersen, Hans von der Maase, Jakob Kjellberg, Helle Timm, Christoffer Johansen

**Affiliations:** 1 Danish Cancer Society Research Center, Copenhagen, Denmark; 2 Research Unit for General Practice, Aarhus University, Aarhus, Denmark; 3 Department of Oncology, Palliative Research Group, Rigshospitalet, Copenhagen University Hospital, Copenhagen, Denmark; 4 Department of Oncology, Rigshospitalet, Copenhagen University Hospital, Copenhagen, Denmark; 5 KORA, The Danish Institute for Local and Regional Government Research, Copenhagen, Denmark; 6 Knowledge Center for Rehabilitation and Palliative Care, University of Southern Denmark, Nyborg, Denmark

## Abstract

**Background:**

Specialised palliative care trials often fail to address intervention effects on caregiver anxiety and depression, particularly in bereavement. We evaluate effects of specialised palliative care and dyadic psychological intervention on caregiver anxiety and depression in a randomised controlled trial (RCT).

**Methods:**

Patients with incurable cancer and limited antineoplastic treatment options and their caregivers, recruited from a university hospital oncology department, were randomised (1:1) to care as usual or accelerated transition from oncological treatment to home-based specialised palliative care. We assessed caregivers’ symptoms of anxiety and depression with the Symptom Checklist-92 up to six months after randomisation and 19 months into bereavement, and estimated intervention effects in mixed effects models.

**Results:**

The ‘Domus’ trial enrolled 258 caregivers. The intervention significantly attenuated increases in caregivers’ symptoms of anxiety overall (estimated difference, −0.12; 95% confidence interval, −0.22 to −0.01, *p* *=* 0.0266), and symptoms of depression at eight weeks (−0.17; −0.33 to −0.02; *p* *=* 0.0314), six months (−0.27; −0.49 to −0.05; *p* *=* 0.0165), and in bereavement at two weeks (−0.28; −0.52 to −0.03; *p* *=* 0.0295) and two months (−0.24; −0.48 to −0.01; *p* *=* 0.0448).

**Conclusions:**

This first RCT evaluating specialised palliative care with dyadic psychological support significantly attenuated caregiver anxiety and depression before and during bereavement. (Clinicaltrials.gov: NCT01885637)

## Introduction

The majority of patients with advanced cancer require substantial support, often provided by spouses or family members at a cost to caregivers’ own mental health. Spouses are at significantly greater risk of antidepressant use and hospitalisation for severe depression than the general population,^1,2^ even in bereavement.^1–3^ Every third to fifth caregiver of patients with advanced cancer experiences elevated symptoms of anxiety or depression.^4^ Patients and caregivers may cope with disease in interaction,^5^ and the way they support each other in coping with cancer affects their distress, supportive care needs, and quality of life.^6–8^ A meta-analysis has documented that intervening at the level of the patient-caregiver dyad significantly improves individual outcomes in both patients and caregivers, as well as their relationship.^9^ Effects of dyadic interventions seem to be equal in size to those of individually focused interventions.^10^ Interventions at the dyad level have the distinct advantage of being able to address dyadic processes, such as common coping efforts or communication, in addition to the individual patient and caregiver. Interventions that lower caregivers’ psychological distress could lead to better support for patients, as well as prevent negative long-term effects for caregivers.

Palliative care aims to alleviate suffering in patients and families.^11^ Still, in eight published randomised controlled trials (RCTs) of out-patient multidisciplinary specialised palliative care identified in PubMed until December 2017, interventions were primarily patient-focused.^12–19^ Only two included well-defined, manualised psychosocial intervention,^15,17^ with one systematically including caregivers.^17^ Only two trials assessed symptoms of anxiety or depression in caregivers,^12,17^ finding effects on one or both,^20,21^ and a single trial assessed depression in bereavement, finding no effect.^22^ Many previous trials included homogenous populations^23^ and caregivers’ symptoms of anxiety or depression have only been assessed in trials with highly educated participants.^20,21^

The ‘Domus’ trial is the first RCT evaluating home-based specialised palliative care with integrated dyadic psychological intervention. It was conducted in a socioeconomically diverse population ensured by a Scandinavian health care setting with equitable access to care. The primary aim was to increase patients’ time at home and the number of home deaths, and the psychological intervention targeted distress in patients and caregivers.^24,25^ We hypothesised that targeting patients’ and caregivers’ distress together could improve outcomes for both. This study examines the effect on the secondary outcomes of caregivers’ symptoms of anxiety and depression.

## Methods

### Study design

The Domus study was a parallel-group RCT, with patients and caregivers recruited from the Department of Oncology at Rigshospitalet, Copenhagen University Hospital, Denmark.^24^ The study protocol was approved by the Danish National Committee on Health Research Ethics (File: 37237) and the Danish Data Protection Agency (File: 2007-58-0015). The trial was registered at clinicaltrials.gov (Identifier: NCT01885637).

### Participants

All potentially eligible patients attending the Department of Oncology at Rigshospitalet, Copenhagen University Hospital were screened for the following eligibility criteria: (1) incurable cancer; (2) limited antineoplastic treatment options or patient chose to forego antineoplastic treatment; (3) living in the Capital Region of Copenhagen; (4) 18 years or older. Limited antineoplastic treatment options were defined for each group of cancers as disease refractory to a specific treatment line, e.g., 3rd line antineoplastic treatment for metastatic breast cancer.^24^ Patients were ineligible if they already received care from a specialised palliative care team, could not be discharged, or were unable to cooperate. Until November 2014, performance status 2–4 was a further inclusion criterion, which was dropped due to slow enrollment. Eligible patients could ask a caregiver, 18 years or older (no other criteria applied), to participate e.g., a partner, adult child, or friend. Both provided written consent.

### Randomisation

Patients and caregivers were assigned to the intervention or care-as-usual control group with a computer generated 1:1 randomisation sequence with varying block size, generated by a statistician not affiliated with the project. Project nurses blinded to block size enrolled and randomised participants using numbered, sealed, and opaque envelopes. As the trial included a behavioral intervention, blinding was not possible.

### Procedure

The design of the Domus intervention,^24^ including the psychological component,^25^ has previously been presented in detail. Briefly, patients and caregivers in the intervention group received an accelerated transition from hospital-based oncological treatment to specialised palliative care at home. The transition included a home-care conference within five working days of randomisation with representatives from one of nine specialised palliative care teams, municipal nursing services, if possible the general practitioner and project psychologist. After the home-care conference, patients received continuing needs-based care based on national guidelines^26^ from their specialised palliative care team, their oncologist, general practitioner, and municipal nursing services. Specialised palliative care teams in Denmark are required to consist of at least two health care professions beside physicians and nurses,^27^ often psychologists or social workers. Five participating multidisciplinary teams in the Domus trial were based at palliative care units in hospitals, four at stand-alone hospices, but all provided specialised palliative home care. A manualised psychological intervention targeted the patient-caregiver dyad, aiming to decrease distress in both patients and caregivers. Two sessions within one month of randomisation were followed by monthly needs-assessment and/or needs-based sessions.^25^ Need for sessions was defined as either the presence of psychiatric disorder or psychological distress that prevented the dyad from adjusting to their situation, or as psychosocial barriers to receiving care, such as disagreements within the dyad, or communication with health care professionals. Sessions could also be planned if the patient or caregiver was at increased risk for future distress or adverse bereavement outcomes. This risk assessment was based on clinical judgment and central literature about risk for distress and adverse bereavement outcomes.^28–32^ After a patient’s death, their caregiver was offered one to two closing sessions. The psychological intervention was dyadic in its target (distress in both dyad members), as well as its primary format (dyadic sessions), and focused on content decided with the dyad.^25^ Sessions were based on existential phenomenological therapy. The existential-phenomenological approach aimed to help dyads adapt more flexibly by exploring alternative ways of relating to their situation and each other and identifying inflexible or rigid aspects of their world-view. For instance, some dyads might not accept help or care, in order to retain a highly valued self-view of independence. Psychologists helped dyads understand their choices within the constraints and possibilities presented by their situation. Dyads received sessions based on the need of each individual dyad, and patients and caregivers could also receive individual sessions, for e.g., patient-specific or caregiver-specific depression. The dyad remained the primary unit of care. Psychologists collaborated with members of the specialised palliative care team as needed.

The control group received care as usual. The Danish health care system is tax-financed and provides free access to healthcare services including general practitioners, general practitioner out-of-hours services, hospital treatment, as well as in-home nursing, home care, and nursing homes. Home-based specialised palliative care is provided by hospital and hospice-based teams, and patients are free to continue oncological treatment alongside specialised palliative care. Some, but not all, specialised palliative care teams include psychologists, and access to psychological support in specialised palliative care is thus not systematic. Care as usual for patients and caregivers randomised to the control group included the possibility of later referral to specialised palliative care, but neither the accelerated transition process, nor the dyadic psychological intervention.

**Fig. 1:**
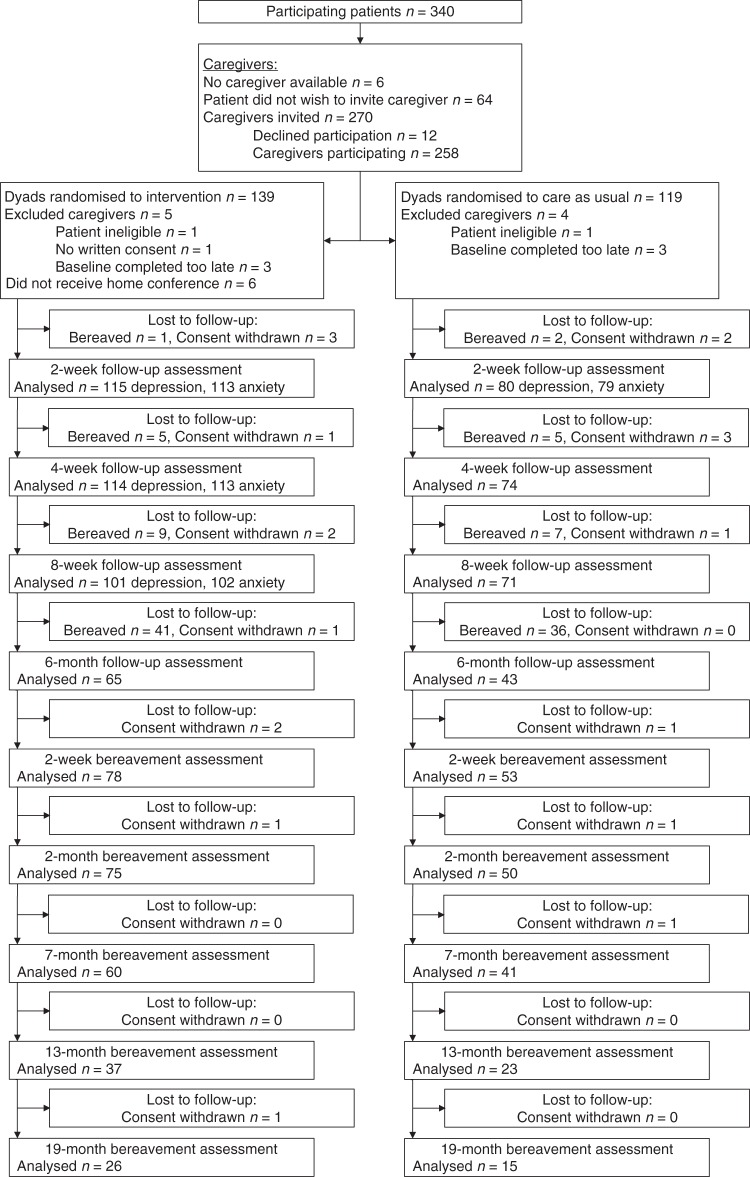
Trial profile of caregivers participating in the Domus study, *n* = 258. Consort flow-chart presenting numbers of caregivers approached for participation, allocated to intervention or control group, excluded from analyses, lost to follow-up due to patient’s death or withdrawn consent, and numbers available for analysis at each follow-up time point

Patients and caregivers completed self-report questionnaires maximally three days before randomisation and four times after randomisation (weeks 2, 4, 8, month 6) (Fig. 1). In addition, caregivers completed questionnaires five times after the patient’s death (week 2, months 2, 7, 13, 19). Questionnaires included the anxiety and depression subscales of the Symptom Checklist-92 (SCL-92), which has been validated in a population-based Danish sample and includes cut-off scores for likely cases.^33,34^ A study presenting patient outcomes is currently in preparation [Nordly et al.: Systematic Fast-Track Transition from Oncological Treatment to Dyadic Specialised Palliative Home Care: DOMUS-A randomised clinical trial].

### Statistical analyses

**Fig. 2:**
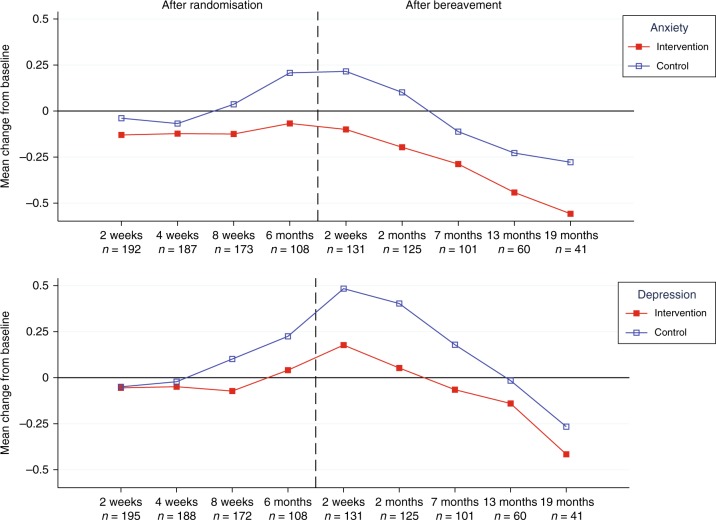
Observed mean change scores in caregivers’ symptoms of anxiety and depression

The target sample size (*n* *=* 380 patients) was determined through power analysis for the primary outcome (patients’ time at home and home deaths) to allow for 10–15% dropout.^24^ Descriptive statistics were calculated for baseline characteristics (Table 1). Mean change scores for anxiety and depression were plotted according to randomisation group and follow-up time (Fig. 2). To investigate the intervention effect on change in symptoms of anxiety and depression, we fitted linear mixed effects models of repeated measures using restricted maximum likelihood based on the intention to treat principle. Degrees of freedom were calculated with the Kenward–Rogers method.^35^ We estimated main intervention effects for change from baseline with 95% confidence intervals (CI), and calculated effect sizes using the standard deviation of the control group at baseline.^36^ Models included fixed effects of caregivers’ age, sex, relationship to the patient (partner, adult child, other), baseline anxiety or depression score, group status (randomisation or control) and follow-up time points (categorical). We included the interaction between follow-up time points and randomisation group to investigate whether effects differed between follow-up times. From this interaction, we estimated intervention effects with 95% CIs and effect sizes for each time point. We tested the fit of covariance structures that took into account varying combinations of correlations within subjects, periods (before and after the patient’s death), and the temporal distance between follow-up times. The best fitting structures, based on Aikaike Information Criteria, were a random subject effect together with an autoregressive AR1 by period (before vs. after the patient’s death) for anxiety, and unstructured for depression. Underlying model assumptions were assessed through visual inspection of residual plots and normal qq-plots. We conducted sensitivity analyses based on two forms of multiple imputation, with 1000 imputations using fully conditional specification.^37^ First, we imputed missing responses on anxiety or depression, unless they were missing in a pre-bereavement assessment because the patient died prior to that assessment. Data were imputed separately for the intervention and the control group, conditional on all nine changes from baseline, baseline observations, age, sex, and caregiver’s relationship to the patient. Second, to simulate a situation in which caregivers’ missing data were related to their levels of anxiety and depression, we shifted all imputed data upward by a value drawn from a normal distribution with mean 0.1 (about one sixth of a standard deviation) and variance 0.005^2^. Supplementary logistic models examined the effect on caregivers’ odds of exceeding cut-off scores for anxiety and depression.^34^ We calculated population average odds ratios using generalised estimation equations with independent working correlation. Models included caregiver sex, age, relation to the patient (partner, adult child, other), baseline anxiety or depression score, group status, and follow-up time (categorical). As in the primary analyses, we included the interaction between follow-up time points and randomisation group to investigate whether effects differed between follow-up times, and estimated effects and effect sizes for each follow-up time point. Analyses were conducted in SAS version 9.4.

**Table 1:** Baseline characteristics of analysed caregivers in the DOMUS study, *n* = 249

	Intervention group *n* = 134*		Control group *n* = 115*
Age, years Mean (SD)	61 (12)		62 (13)
Sex *n* (%)			
Male	49 (37)		40 (39)
Female	85 (63)		75 (65)
Marital status *n* (%)			
Married/cohabiting	123 (92)		103 (90)
Single	7 (5)		7 (6)
Divorced	1 (2)		3 (3)
Widow(er)	-		2 (2)
Missing information	3 (2)		-
Children *n* (%)			
Children	110 (82)		97 (84)
Living at home^**^	*27 (20)*		*24 (21)*
Not living at home^**^	*86 (64)*		*78 (68)*
No children	19 (14)		17 (15)
Missing information	5 (3)		1 (1)
Highest achieved education *n* (%)			
Element./middle school (9 years)	14 (10)		14 (12)
Vocational	35 (26)		31 (27)
High school	2 (2)		2 (2)
Further education (-4½ years)	48 (36)		47 (41)
Higher education (5- years)	27 (20)		16 (14)
Missing information	8 (6)		5 (4)
Relationship to patient *n* (%)			
Spouse/partner	103 (77)		92 (80)
Son/daughter	24 (18)		10 (9)
Other	7 (5)		13 (11)
Cohabiting with patient *n* (%)			
Yes	103 (77)		91 (79)
No	25 (19)		22 (19)
Missing information	6 (5)		2 (2)
Length of relationship with patient, years Mean (SD, range)	38 (15, 5–63)		38 (16, 2–64)
Missing information	5		3
Patient’s cancer diagnosis *n* (%)			
Breast	5 (4)	7 (6)	
CNS	16 (12)	21 (18)	
Connective tissue	5 (4)	8 (7)	
Female genitalia	18 (13)	13 (11)	
Head and neck	6 (5)	9 (8)	
Lower gastrointestinal	15 (11)	13 (11)	
Lung	28 (21)	25 (22)	
Other	11 (8)	1 (1)	
Prostate	17 (13)	5 (4)	
Upper gastrointestinal	13 (10)	13 (11)	
Performance status *n* (%)			
0–1	68 (51)	59 (51)	
2–3	66 (49)	56 (49)	
Baseline anxiety symptoms Mean (SD)	1.00 (0.66)	0.94 (0.66)	
% scoring above cut-off	28	27	
Baseline depression symptoms Mean (SD)	0.84 (0.69)	0.80 (0.64)	
% scoring above cut-off	24	23	

^*^Some percentages do not add up to 100 due to rounding.

^**^Categories are not exclusive

## Results

From June 19, 2013 to August 22, 2016, 340 patients were recruited, of whom 258 (76%) participated with a caregiver. Inclusion was terminated early due to lower than expected drop-out. One hundred thirty-nine dyads were allocated to the intervention, 119 to the control group (Fig. 1). Almost all invited caregivers (96%) participated. We excluded nine caregivers from the present analyses: two patients did not fulfill eligibility criteria, one caregiver did not provide written consent, and six caregivers failed to complete baseline assessments before randomisation (Fig. 1). Eleven caregivers in the intervention and nine in the control group withdrew consent during follow-up and were excluded from analyses subsequently. Participants were not required to give reasons for withdrawing consent, but those recorded related to the patient’s condition, randomisation to the control group, disappointment with the intervention, not wishing to complete questionnaires, or moving out of the Capital Region. Within six months of randomisation, 56 patients (42%) in the intervention group and 50 patients (43%) in the control group died (Fig. 1). During the period of follow-up for this study (until 22 February 2017), a total of 105 (78%) patients in the intervention group and 89 (77%) in the control group had died. At assessments during caregiving, the SCL-92 was completed by between 80 and 84% of available caregivers, who were neither bereaved nor had withdrawn consent. At assessments in bereavement, between 57 and 68% of caregivers completed the measure. Three caregivers were not included in the primary analysis due to missing data on symptoms of anxiety and depression at baseline.

Six dyads in the intervention group did not receive the planned home conference. The number of subsequent visits from the specialised palliative care team differed based on needs. On average, dyads received four psychological intervention sessions before the patient’s death (interquartile range 2 to 6), and 0.4 in bereavement (interquartile range 0 to 1). The majority (63%) of sessions were attended by the patient and caregiver together, and the average number of sessions per month participation in the RCT was 0.8. Of patients in the control group (including patients participating alone), 60% received specialised palliative care, beginning on average 110 days later than the intervention group (Nordly et al. in prep.). Use of psychologists by participants in the control group was not recorded.

Most participating caregivers were patients’ partners and the majority women (Table 1). At baseline about one fourth of caregivers in the intervention and control group exceeded cut-off scores for anxiety and depression (online figs. S1, S2). Caregivers in the intervention group reported more beneficial changes in mean scores for symptoms of anxiety and depression throughout follow-up than caregivers in the control group (Fig. 2).

### Intervention effects on anxiety

**Fig. 3:**
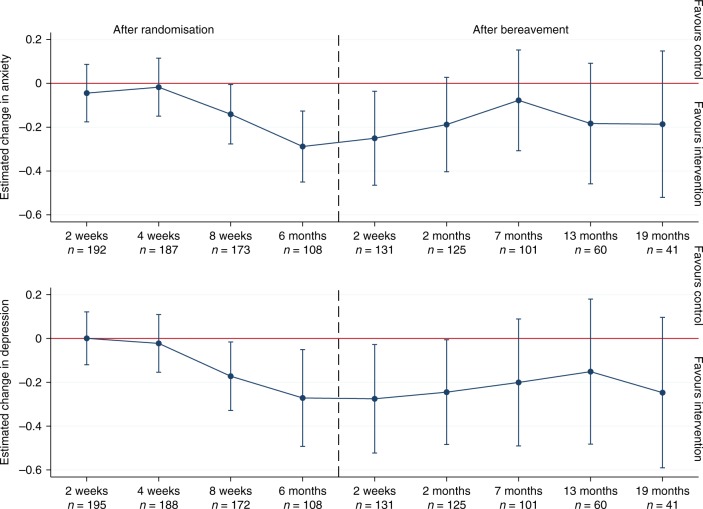
Estimated difference in change in symptoms of anxiety and depression and 95% confidence intervals between caregivers in the intervention and control group. Estimates adjusted for age, sex, relationship to the patient (spouse, adult child, other), baseline score

**Fig. 4:**
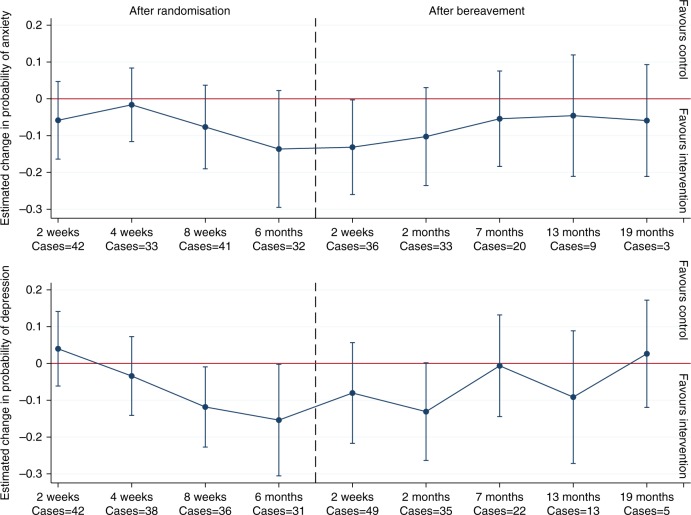
Estimated change in probability of scoring above cut-offs (cases) for anxiety and depression for caregivers (*n* = 41−246) in the intervention compared to the control group. Estimates adjusted for age, sex, relationship to the patient (spouse, adult child, other), baseline score. Probabilities are derived from logistic regression models for anxiety and depression

Mixed effects models estimated that caregivers in the intervention group experienced significantly lower symptoms of anxiety throughout follow-up (estimated difference −0.12; 95% CI, −0.22 to −0.01; *p* = 0.0266; Cohen’s d, −0.19), at eight weeks (−0.14; −0.28 to −0.01; *p* = 0.0421; Cohen’s d, −0.22) and six months (−0.29; −0.45 to −0.13; *p* = 0.0005; Cohen’s d, −0.45) after randomisation, and two weeks into bereavement (−0.25; −0.47 to −0.04; *p* = 0.0223; Cohen’s d, −0.39) (Fig. 3, online Table S1). In models for dichotomised scores, caregivers in the intervention group were significantly less likely than caregivers in the control group to score above the cut-off for anxiety throughout follow-up (OR 0.55; 95% CI, 0.39 to 0.78) (Fig. 4, online Table S3).

### Intervention effects on depression

We found no significant overall intervention effect for symptoms of depression (−0.06; 95% CI, −0.17 to 0.05; *p* *=* 0.2992; Cohen’s d, −0.09), but caregivers in the intervention group experienced significantly lower symptoms than caregivers in the control group at eight weeks (−0.17; -0.33 to −0.02; *p* *=* 0.0314; Cohen’s d, −0.26) and six months after randomisation (−0.27; −0.49 to −0.05; *p* *=* 0.0165; Cohen’s d, −0.41), as well as two weeks (−0.28; −0.52 to −0.03; *p* *=* 0.0295; Cohen’s d, −0.42) and two months (−0.24; −0.48 to −0.01; *p* *=* 0.0448; Cohen’s d, −0.37) after the patient’s death (online Table S2). In models for dichotomised scores, caregivers in the intervention group were significantly less likely than caregivers in the control group to score above the cut-off for depression eight weeks (OR 0.4; 0.17 to 0.92) and six months after randomisation (OR 0.38; 0.14 to 0.98) (Fig. 4, online Table S3).

### Sensitivity analyses

Sensitivity analyses using multiple imputation did not change conclusions (online Table S1, S2), regardless of the method of imputation.

## Discussion

We found significantly attenuated increases in symptoms of anxiety and depression in caregivers in the intervention group compared to caregivers in the control group both before and after the patient’s death. Differences reached significance from eight weeks after randomisation to two months after the patient died, as well as for the main effect on symptoms of anxiety.

Our study is the first to demonstrate effects of specialised palliative care with dyadic psychological intervention on caregivers’ symptoms of anxiety and depression both before and after the patient’s death. We found small to medium effect sizes (0.26 to 0.42 for depression, 0.19 to 0.45 for anxiety, online Table S1 and S2), which is comparable to effect sizes reported in previous trials assessing effects on caregiver anxiety and/or depression (0.30 to 0.39).^20,21^ The significantly reduced likelihood of intervention group caregivers exceeding cut-of scores for anxiety and depression at several follow-up points indicates that effects are clinically significant.

Caregivers are at short-term and long-term risk of diminished mental health,^3,4^ but may not feel entitled to seek support.^38^ Although the interactions between follow-up time points and group status in mixed effects models were not significant, our results yield a pattern of increasing effects with time until the six-month follow-up, significant from eight weeks after randomisation. This indicates that alleviating caregivers’ symptoms of anxiety and depression may require continued needs assessments and intervention over time. It may also reflect greater strain on caregivers, and therefore potential to intervene, in the time surrounding patients’ death. Palliative care clinicians see many caregivers through their involvement in patients’ care and may be uniquely positioned to refer caregivers to services to prevent mental health problems. Several efficacious caregiver interventions exist,^39^ but interventions in RCTs of specialised palliative care focus predominantly on patients. Neither of the previous trials assessing caregivers’ symptoms of anxiety and depression focused on support for the patient-caregiver dyad together,^20,21^ and one explicitly separated patient and caregiver psychosocial interventions to encourage disclosure of sensitive topics.^21^ The similar effect sizes found in our study mostly targeting distress in patients and caregivers together indicates that dyadic interventions may also be appropriate.

The Domus RCT tested a complex intervention and effects cannot be attributed to specific intervention components. This mirrors the nature of specialised palliative care, where multidisciplinary management is central,^11^ and complex trials are crucial to solidify the evidence base. The Domus intervention may have affected caregiver distress through multiple pathways, lowering distress directly, indirectly through intervention effects on patients, or through dyadic effects. Examples of such pathways could be (a) direct: lowering caregivers’ depression by helping them relate to the weakened patient in new ways or providing them with knowledge about symptom management leading to increased feelings of mastery and diminished anxiety, (b) indirect: reducing patients’ physical or psychological symptoms, leading to lower caregiver burden, or (c) dyadic: facilitating communication about wishes for professional support, leading to increased acceptance of care and lower caregiver strain. Although interventions targeting the dyad often result in effect-sizes equal to those of interventions targeting patients or caregivers alone,^10^ we believe that intervening at the level of the dyad has certain advantages. As psychological distress is interrelated between patients and caregivers^40,41^ and, at least in part, dependent on the way members of the dyad cope together,^6^ dyadic interventions may be better positioned to create and maintain change than efforts where only one partner is receiving the intervention. Positive effects of individual interventions in one partner might be countered by unchanged or worsening distress in the other, not receiving the intervention. Dyadic intervention also allows clinicians to address issues that are relational in nature, e.g., the patient-caregiver dyad’s communication. Further, the needs of patients and caregivers may not always coincide, and dyads may disagree on issues such as how much information to give their families, how much help to receive from health care professionals, or where they would like the patient to spend the end of life. In dyadic interventions, the clinician can directly facilitate conversation about such issues. Future investigations to identify specific mechanisms and their optimal timing and delivery (to the patient or caregiver alone or together) could further strengthen effective components of complex specialised palliative care interventions.

Among the strengths of this study is the inclusion of a manualised psychological intervention^25^ to ensure that all sessions were based on the same principles and methods. The manual provides a description of the intervention rationale and delivery and can thus inform clinical practice and future research. Previous trials assessing caregiver anxiety or depression were conducted mostly with highly educated populations,^20,21^ biased due to their socioeconomic resources, and the findings cannot be directly generalised to more diverse populations of caregivers. The Domus study was conducted in a Scandinavian health care setting that ensures access and equitable treatment and affords the opportunity to reach patients and caregivers across socioeconomic positions. As a result, more than one third of participating caregivers had less than high school education and our results may be generalised to socioeconomically diverse caregiver populations. On the other hand, the universal health care coverage in Denmark limits direct generalisability to health care settings with lower access to palliative care and mental health. However, in such settings the effect of the intervention might have been even greater. Systematic screening for inclusion of all cancer patients attending the Department of Oncology, Rigshospitalet, Copenhagen University Hospital ensured that all potentially eligible patients were approached and informed of the study and the very high participation rate among invited caregivers (96%) increases our confidence in the generalisability of effects. Our previous investigation of uptake of the psychological intervention component indicated that the intervention was feasible and acceptable to patients and caregivers.^25^

This study is limited by the decreasing number of respondents in bereavement, as some caregivers were not, or only recently, bereaved at the time of analyses. However, sensitivity analyses yielded very similar results. Eight percent of caregivers withdrew consent at some point during follow-up. This number is only slightly larger than that reported in one of the previous studies assessing caregiver anxiety and depression (4%),^20^ considering the longer follow-up in the Domus trial, lasting up to 19 months after the patients’ death. Like previous studies, our sample was recruited in a single location. However, nine different palliative care teams provided the intervention, limiting the effect of the single recruitment site on generalisability. The lack of an established minimal clinically important difference (MCID) on the SCL-92 prevents us from conclusively identifying the statistically significant changes as clinically significant as well. However, we used the best available approximation of clinical significance, the cut-off scores determined specifically for the Danish population. Results from the dichotomised analyses based on these cut-off scores serve to indicate that our findings have clinical relevance. Finally, we did not measure intervention adherence, limiting the confidence with which we can attribute effects to specific intervention components. However, psychologists participated in biweekly group-supervision to support the uniform implementation of the psychological intervention component.

We have demonstrated that an accelerated transition to home-based specialised palliative care in combination with dyadic psychological intervention significantly improved caregivers’ symptoms of anxiety and depression, both before and after the patient’s death. The Domus RCT is the first trial of home-based specialised palliative care to include a manualised psychological intervention that targets the patient-caregiver dyad as the unit of care. Targeting distress in caregivers not only improves their mental health, but may also counteract other negative effects of caregiving, such as increased health care use and work impairments,^42^ creating a ripple of public health and societal impacts both during caregiving and in bereavement.

## Electronic supplementary material

Figure S1. Observed proportion of caregivers scoring above cut-off scores for anxiety

Figure S2. Observed proportion of caregivers scoring above cut-off scores for depression

Table S1. Estimated differences in change scores from baseline for anxiety between caregivers in the intervention and control group (Online only)

Table S2. Estimated differences in change scores from baseline for depression between caregivers in the intervention and control group (Online only)

Table S3. Estimated odds ratios for caregivers in the intervention compared to the control group of scoring above cut-offs (cases) for anxiety and depression (Online only)

Color Artwork Form

## **Availability of data and material:**

The data that support the findings of this study are available on reasonable request from the corresponding author (A.H.). The data are not publicly available due to participant privacy and consent.
