# Supplementary figures and images for: Effect of home-based specialised palliative care and dyadic psychological intervention on caregiver anxiety and depression: a randomised controlled trial
Source: Br J Cancer. 2018 Nov 14;119(11):1307–15. doi: 10.1038/s41416-018-0193-8 (PMC6265292; doi:10.1038/s41416-018-0193-8)

Figure S1. Observed proportion of caregivers scoring above cut-off scores for anxiety

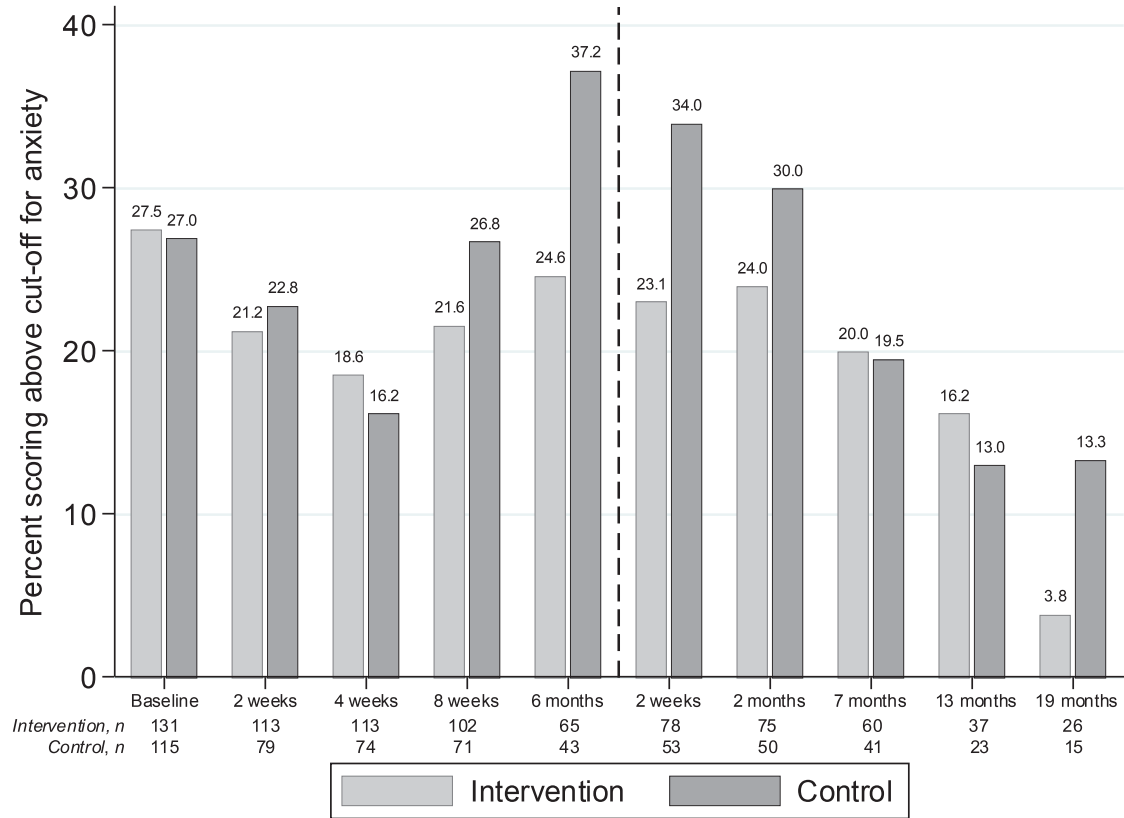

Supplement: Supplementary file 1 — Figure S1. Observed proportion of caregivers scoring above cut-off scores for anxiety [file 41416_2018_193_MOESM1_ESM.pdf]

Figure S2. Observed proportion of caregivers scoring above cut-off scores for depression

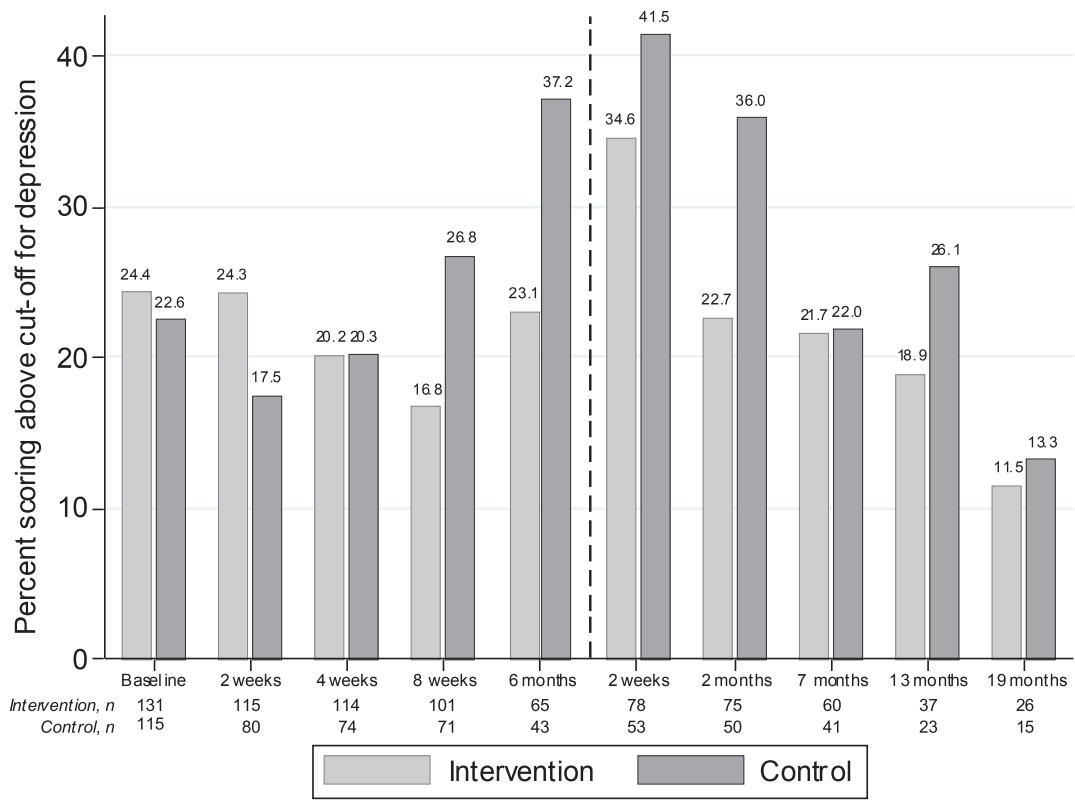

Supplement: Supplementary file 2 — Figure S2. Observed proportion of caregivers scoring above cut-off scores for depression [file 41416_2018_193_MOESM2_ESM.pdf]
