# Supplementary material for: Effect of home-based specialised palliative care and dyadic psychological intervention on caregiver anxiety and depression: a randomised controlled trial
Source: Br J Cancer. 2018 Nov 14;119(11):1307–15. doi: 10.1038/s41416-018-0193-8 (PMC6265292; doi:10.1038/s41416-018-0193-8)
Supplement: Supplementary file 3 — Table S1. Estimated differences in change scores from baseline for anxiety between caregivers in the intervention and control group (Online only) [file 41416_2018_193_MOESM3_ESM.pdf]

Table S1. Estimated differences in change scores from baseline for anxiety between caregivers in the intervention and control group (Online only)

Follow-up time point	*n*	Mixed model results		Multiple imputation	Multiple imputation, shifted values	Raw mean change score		Estimated mean change score	
		**Mean difference (95% CI)**	**Cohen's d**	**Mean difference (95% CI)**	**Mean difference (95% CI)**	**Intervention group** *(mean (SD))*	**Control group** *(mean (SD))*	**Intervention group (95% CI)**	**Control group (95% CI)**
2 weeks	192	-0.04 (-0.18; 0.09)	-0.07	-0.07 (-0.22; 0.08)	-0.28 (-0.55; -0.02)	-0.13 (0.39)	-0.04 (0.38)	-0.10 (-0.21; 0.01)	-0.05 (-0.18; 0.07)
4 weeks	187	-0.02 (-0.15; 0.12)	-0.03	-0.02 (-0.17; 0.13)	-0.21 (-0.46; 0.03)	-0.12 (0.48)	-0.07 (0.36)	-0.10 (-0.21; 0.01)	-0.08 (-0.21; 0.04)
8 weeks	173	-0.14 (-0.28; -0.01)	-0.22	-0.20 (-0.36; -0.03)	-0.46 (-0.72; -0.20)	-0.12 (0.47)	0.04 (0.56)	-0.10 (-0.21; 0.01)	0.04 (-0.08; 0.17)
6 months	108	-0.29 (-0.45; -0.13)	-0.45	-0.35 (-0.61; -0.09)	-0.58 (-1.11; -0.04)	-0.08 (0.44)	0.21 (0.74)	-0.04(-0.17; 0.09)	0.25 (0.10; 0.39)
**Bereavement follow-up**									
2 weeks	131	-0.25 (-0.47; -0.04)	-0.39	-0.22 (-0.57; 0.12)	-0.90 (-1.64; -0.17)	-0.10 (0.65)	0.22 (0.85)	-0.02 (-0.18; 0.13)	0.23 (0.05; 0.41)
2 months	125	-0.19 (-0.40; 0.03)	-0.29	-0.16 (-0.48; 0.17)	-0.86 (-1.55; -0.16)	-0.20 (0.56)	0.10 (0.78)	-0.13 (-0.29; 0.02)	0.05 (-0.13; 0.23)
7 months	101	-0.08 (-0.31; 0.15)	-0.12	-0.05 (-0.37; 0.28)	-0.63 (-1.32; 0.07)	-0.29 (0.61)	-0.11 (0.85)	-0.20 (-0.36; -0.03)	-0.12 (-0.31; 0.07)
13 months	60	-0.18 (-0.46; 0.09)	-0.29	-0.06 (-0.43; 0.32)	-0.13 (-1.32; 1.06)	-0.44 (0.59)	-0.23 (0.74)	-0.37 (-0.55; -0.18)	-0.18 (-0.41; 0.04)
19 months	41	-0.19 (-0.52; 0.15)	-0.29	-0.16 (-1.08; 0.76)	-0.83 (-7.34; 5.68)	-0.56 (0.60)	-0.28 (0.72)	-0.48 (-0.70; -0.27)	-0.30 (-0.57; -0.02)
**Main effect of randomization**	246	-0.12 (-0.22; -0.01)	-0.19	-0.16 (-0.30; -0.03)	-0.42 (-0.68; -0.16)				
**Test for interaction*:**		F(8, 562) = 1.82 p = 0.0705							

* Interaction of follow-up time point (categorical) and randomization group

The main effect of time was not estimated, as this was not the main interest.
