# Supplementary material for: Effect of home-based specialised palliative care and dyadic psychological intervention on caregiver anxiety and depression: a randomised controlled trial
Source: Br J Cancer. 2018 Nov 14;119(11):1307–15. doi: 10.1038/s41416-018-0193-8 (PMC6265292; doi:10.1038/s41416-018-0193-8)
Supplement: Supplementary file 4 — Table S2. Estimated differences in change scores from baseline for depression between caregivers in the intervention and control group (Online only) [file 41416_2018_193_MOESM4_ESM.pdf]

Table S2. **Estimated differences in change scores from baseline for depression between caregivers in the intervention and control group (Online only)**

Follow-up time point	*n*	Mixed model results: Mean difference (95% CI)	Mixed model results: Cohen's d	Multiple imputation: Mean difference (95% CI)	Multiple imputation, shifted values: Mean difference (95% CI)	Raw mean change score: Intervention group *(mean (SD))*	Raw mean change score: Control group *(mean (SD))*	Estimated mean change score: Intervention group (95% CI)	Estimated mean change score: Control group (95% CI)
2 weeks	195	0.00 (-0.12; 0.12)	0.00	-0.01 (-0.13; 0.11)	-0.20 (-0.46; 0.06)	-0.06 (0.42)	-0.05 (0.41)	-0.07 (-0.18; 0.04)	-0.07 (-0.20; 0.05)
4 weeks	188	-0.02 (-0.15; 0.11)	-0.03	-0.03 (-0.17; 0.11)	-0.21 (-0.47; 0.05)	-0.05 (0.48)	-0.02 (0.43)	-0.07 (-0.18; 0.04)	-0.05 (-0.18; 0.08)
8 weeks	172	-0.17 (-0.33; -0.02)	-0.26	-0.19 (-0.36; -0.02)	-0.49 (-0.78; -0.20)	-0.07 (0.46)	0.10 (0.66)	-0.08 (-0.21; 0.04)	0.09 (-0.06; 0.23)
6 months	108	-0.27 (-0.49; -0.05)	-0.41	-0.30 (-0.55; -0.05)	-0.44 (-0.91; 0.03)	0.04 (0.53)	0.22 (0.69)	0.02 (-0.14; 0.18)	0.29 (0.10; 0.48)
**Bereavement follow-up**									
2 weeks	131	-0.28 (-0.52; -0.03)	-0.42	-0.25 (-0.55; 0.05)	-1.00 (-1.51; -0.49)	0.18 (0.65)	0.48 (0.85)	0.19 (0.01; 0.37)	0.47 (0.26; 0.67)
2 months	125	-0.24 (-0.48; -0.01)	-0.37	-0.24 (-0.52; 0.04)	-0.89 (-1.40; -0.38)	0.05 (0.59)	0.40 (0.93)	0.06 (-0.11; 0.23)	0.31 (0.11; 0.51)
7 months	101	-0.20 (-0.49; 0.09)	-0.30	-0.14 (-0.47; 0.18)	-0.52 (-1.13; 0.10)	-0.07 (0.79)	0.18 (0.91)	-0.06 (-0.26; 0.15)	0.14 (-0.09; 0.38)
13 months	60	-0.15 (-0.48; 0.18)	-0.23	-0.03 (-0.40; 0.34)	-0.51 (-1.59; 0.58)	-0.14 (0.80)	-0.02 (0.88)	-0.15 (-0.38; 0.07)	-0.00 (-0.27; 0.26)
19 months	41	-0.25 (-0.59; 0.10)	-0.37	-0.12 (-1.55; 1.31)	0.15 (-4.37; 4.67)	-0.42 (0.70)	-0.27 (0.75)	-0.40 (-0.63; -0.18)	-0.16 (-0.43; 0.12)
**Main effect of randomization**	246	-0.06 (-0.17; 0.05)	-0.09	-0.04 (-0.19; 0.11)	-0.14 (-0.41; 0.12)				
**Test for interaction*:**		F(8, 87.5) = 1.73 p = 0.1022							

* Interaction of follow-up time point (categorical) and randomization group

The main effect of time was not estimated, as this was not the main interest.
