# Supplementary material for: Effect of home-based specialised palliative care and dyadic psychological intervention on caregiver anxiety and depression: a randomised controlled trial
Source: Br J Cancer. 2018 Nov 14;119(11):1307–15. doi: 10.1038/s41416-018-0193-8 (PMC6265292; doi:10.1038/s41416-018-0193-8)
Supplement: Supplementary file 5 — Table S3. Estimated odds ratios for caregivers in the intervention compared to the control group of scoring above cut-offs (cases) for anxiety and depression (Online only) [file 41416_2018_193_MOESM5_ESM.pdf]

Table S3. Estimated odds ratios for caregivers in the intervention compared to the control group of scoring above cut-offs (cases) for anxiety and depression (Online only)

Follow-up time point	Anxiety		Depression	
	n	**OR* (95% CI)**	***n***	**OR* (95% CI)**
2 weeks	192	0.63 (0.28; 1.44)	195	1.37 (0.61; 3.11)
4 weeks	187	0.86 (0.35; 2.11)	188	0.77 (0.34; 1.75)
8 weeks	173	0.57 (0.25; 1.30)	172	0.40 (0.17; 0.92)
6 months	108	0.43 (0.16; 1.14)	108	0.38 (0.14; 0.98)
**Bereavement follow-up**				
2 weeks	131	0.38 (0.15; 0.97)	131	0.62 (0.27; 1.40)
2 months	125	0.48 (0.19; 1.23)	125	0.42 (0.17; 1.00)
7 months	101	0.61 (0.19; 1.96)	101	0.95 (0.33; 2.78)
13 months	60	0.60 (0.11; 3.45)	60	0.50 (0.13; 1.90)
19 months	41	0.28 (0.01; 5.48)	41	1.40 (0.21; 9.35)
**Test for interaction†**	Chi2(8) = 2.25, p = 0.97		Chi2(8) = 10.36, p = 0.24	
**Main effect of randomization**	246	0.55 (0.39; 0.78)	246	0.65 (0.40; 1.07)

* Reference: control group.

† Interaction of follow-up time point (categorical) and randomization group

The main effect of time was not estimated, as this was not the main interest.
