# Supplementary material for: Effect of home-based specialised palliative care and dyadic psychological intervention on caregiver anxiety and depression: a randomised controlled trial
Source: Br J Cancer. 2018 Nov 14;119(11):1307–15. doi: 10.1038/s41416-018-0193-8 (PMC6265292; doi:10.1038/s41416-018-0193-8)
Supplement: Supplementary file 6 — Color Artwork Form [file 41416_2018_193_MOESM6_ESM.docx]

**Supplementary figures: 2**

Figure S1 – Caption: Observed proportion of caregivers scoring above cut-off scores for anxiety

Figure S2 – Caption: Observed proportion of caregivers scoring above cut-off scores for depression

**Supplementary tables: 3**

Table S1 – Estimated differences in change scores for anxiety between caregivers in the intervention and control group (Online only)

Legend:

* Interaction of follow-up time point (categorical) and randomization group

The main effect of time was not estimated, as this was not the main interest.

Table S2 – Estimated differences in change scores for depression between caregivers in the intervention and control group (Online only)

Legend:

* Interaction of follow-up time point (categorical) and randomization group

The main effect of time was not estimated, as this was not the main interest.

Table S3 – Estimated odds ratios for caregivers of scoring above cut-offs (cases) for anxiety and depression

Legend:

* Reference: control group

† Interaction of follow-up time point (categorical) and randomization group

The main effect of time was not estimated, as this was not the main interest.
